# Supplementary material for: Causal Relationship between Aging and Anorexia Nervosa: A White-Matter-Microstructure-Mediated Mendelian Randomization Analysis
Source: Biomedicines. 2024 Aug 16;12(8):1874. doi: 10.3390/biomedicines12081874 (PMC11351342; doi:10.3390/biomedicines12081874)
Supplement: Supplementary file 1 [file biomedicines-12-01874-s001.zip › Supplementary Figure/Figure S9/Leaveoneout plot of MR analysis for ubm-b-1766 to AN.pdf]

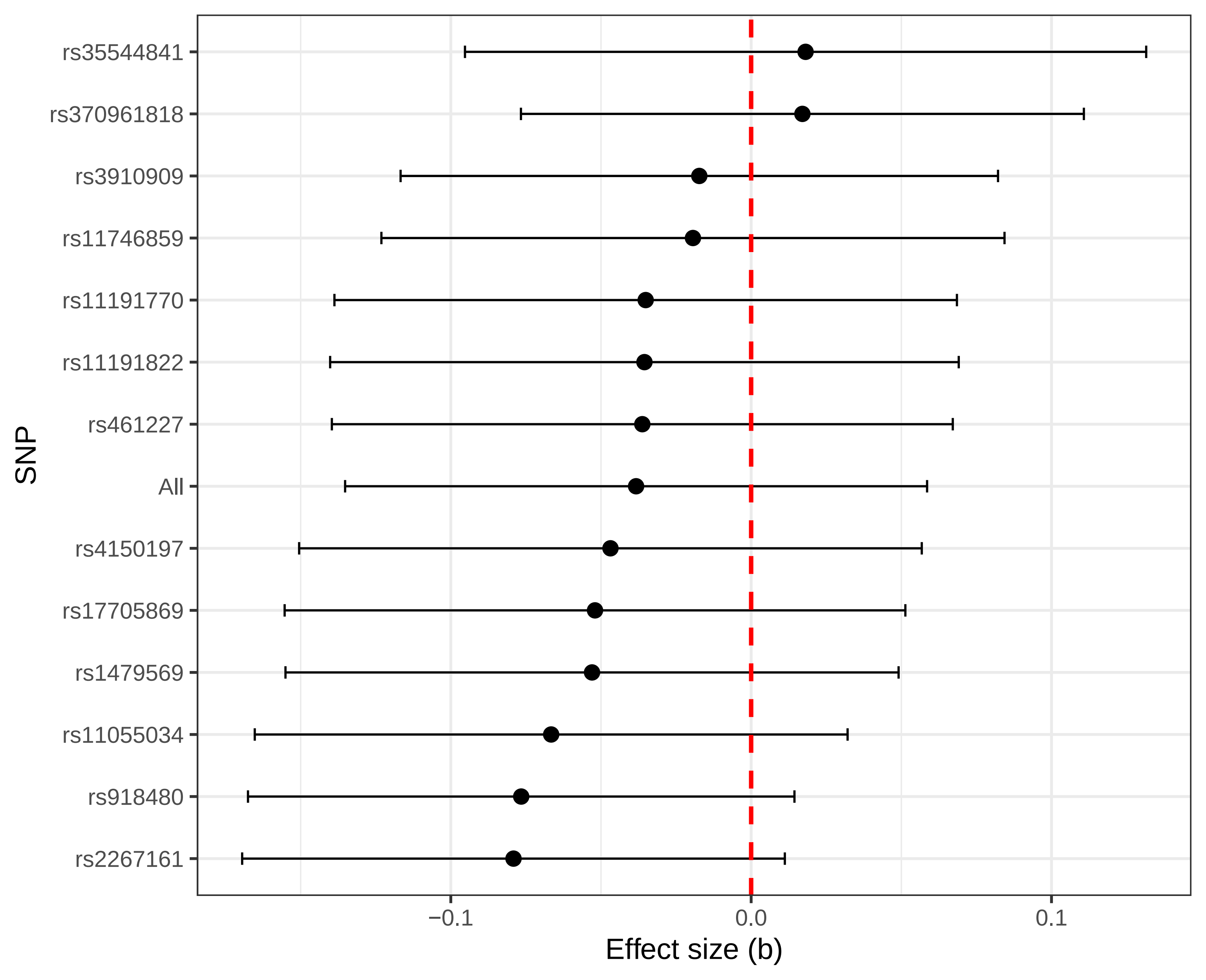
rs35544841
rs370961818
rs3910909
rs11746859
rs11191770
rs11191822
rs461227
All
rs4150197
rs17705869
rs1479569
rs11055034
rs918480
rs2267161
SNP
−0.1
0.0
0.1
Effect size (b)
